# Supplementary material for: Reversible intercalation of methyl viologen as a dicationic charge carrier in aqueous batteries
Source: Nat Commun. 2019 Jul 19;10:3227. doi: 10.1038/s41467-019-11218-5 (PMC6642176; doi:10.1038/s41467-019-11218-5)
Supplement: Supplementary file 1 — Supplementary Information [file 41467_2019_11218_MOESM1_ESM.pdf]

Supplementary Information

Reversible Intercalation of Large Methyl Viologen as a Dicationic Charge Carrier in Aqueous Batteries

Wei et al.

Supplementary Figures

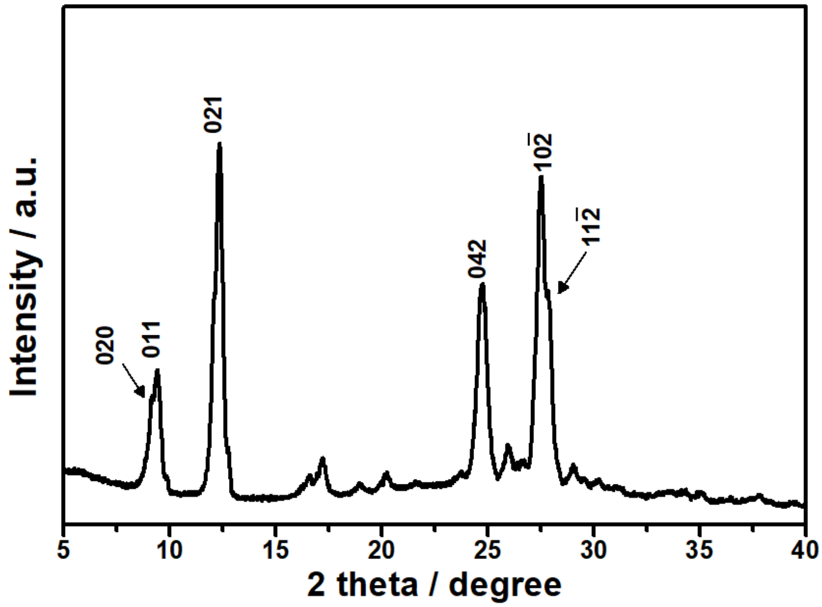

Supplementary Figure 1. The XRD pattern of PTCDA powder.

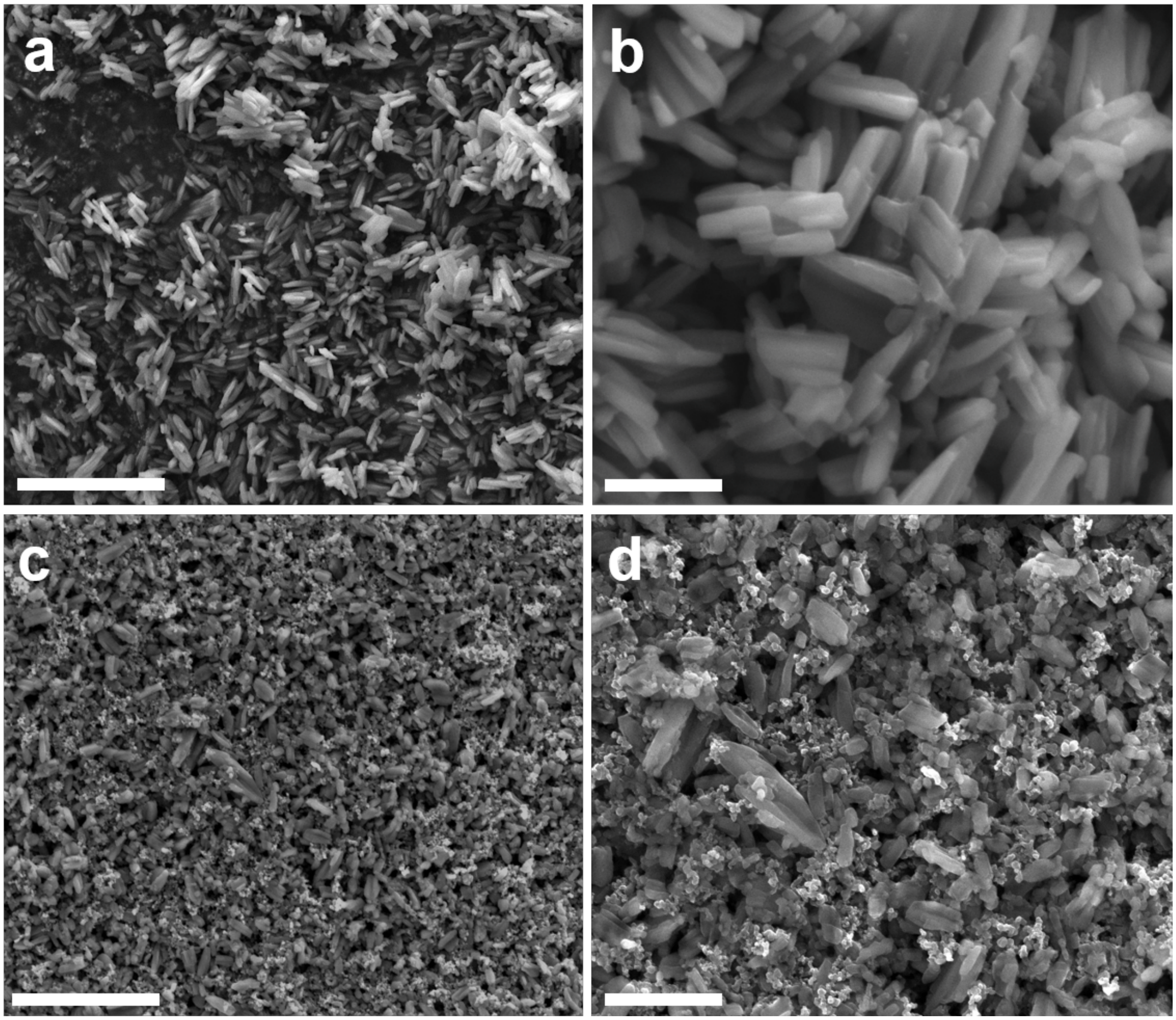

Supplementary Figure 2. Morphological characterization of PTCDA. SEM images of (a, b) PTCDA powder and (c, d) A pristine electrode at the OCV state. Scale bars: (a) 5μm, (b) 1μm, (c) 5μm and (d) 2μm.

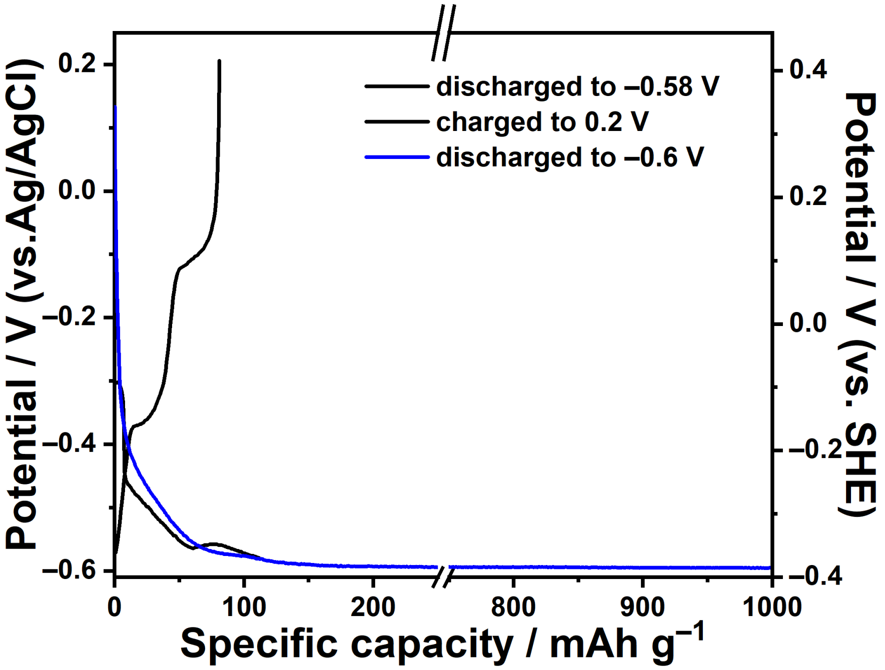

Supplementary Figure 3. Charge/discharge potential profiles in two the potential windows: 0.2~–0.58 V *vs.* Ag/AgCl (black) and 0.2~–0.6 V *vs.* Ag/AgCl (blue).

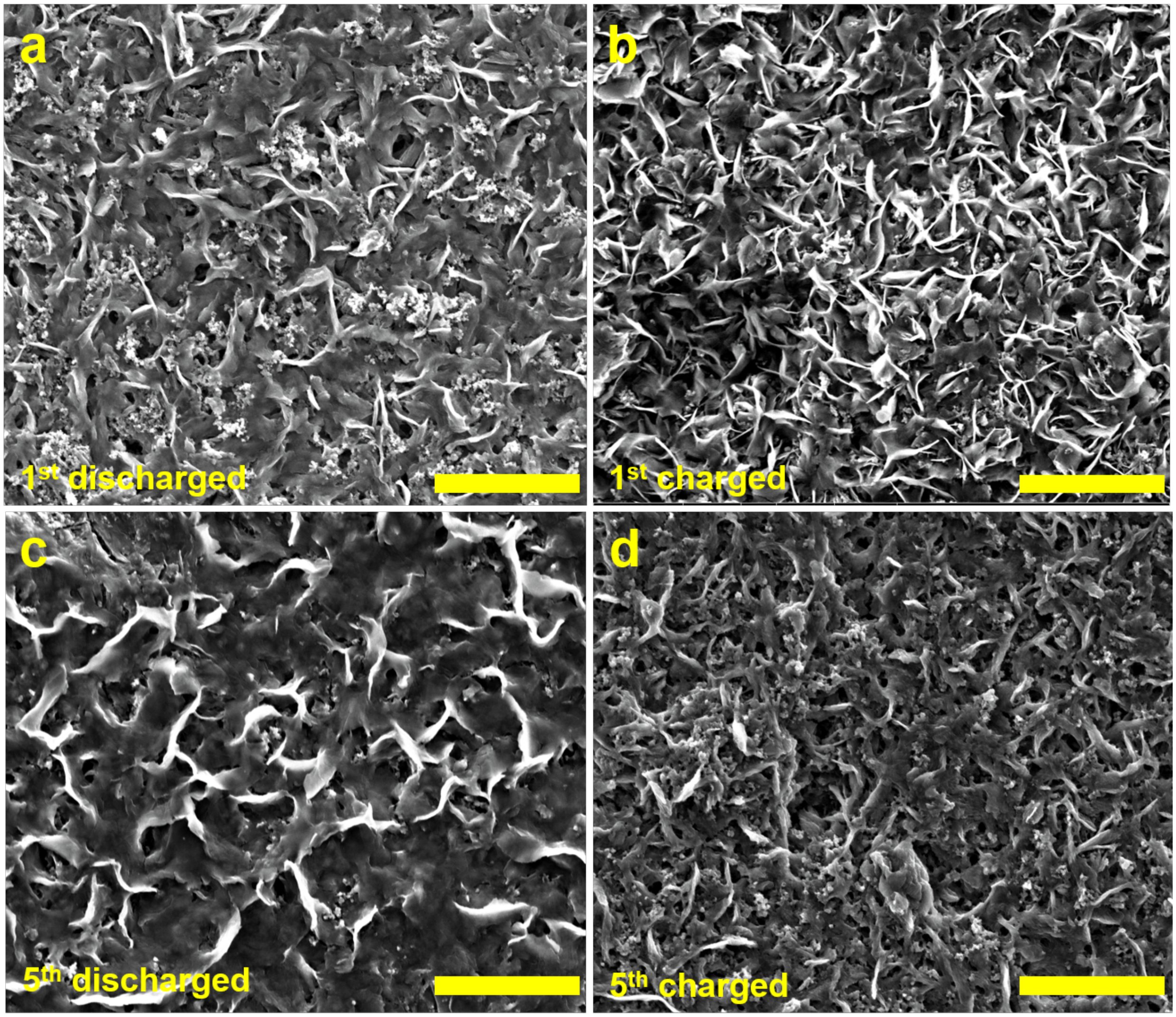

Supplementary Figure 4. ***Ex situ* SEM images for the PTCDA electrode at different state of charge.** (a, b) The 1st cycle; (c, d) The 5th cycle. Scale bars: (a-d) 5μm.

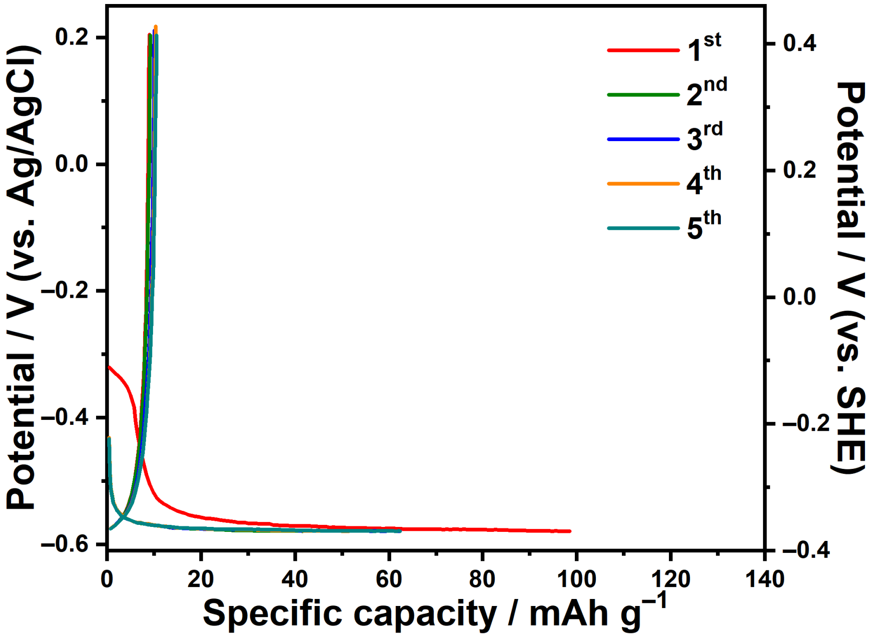

Supplementary Figure 5. GCD curves of a carbon fiber paper tested between –0.58 and 0.2 V *vs.* Ag/AgCl at a current rate of 100 mA g^{-1}.

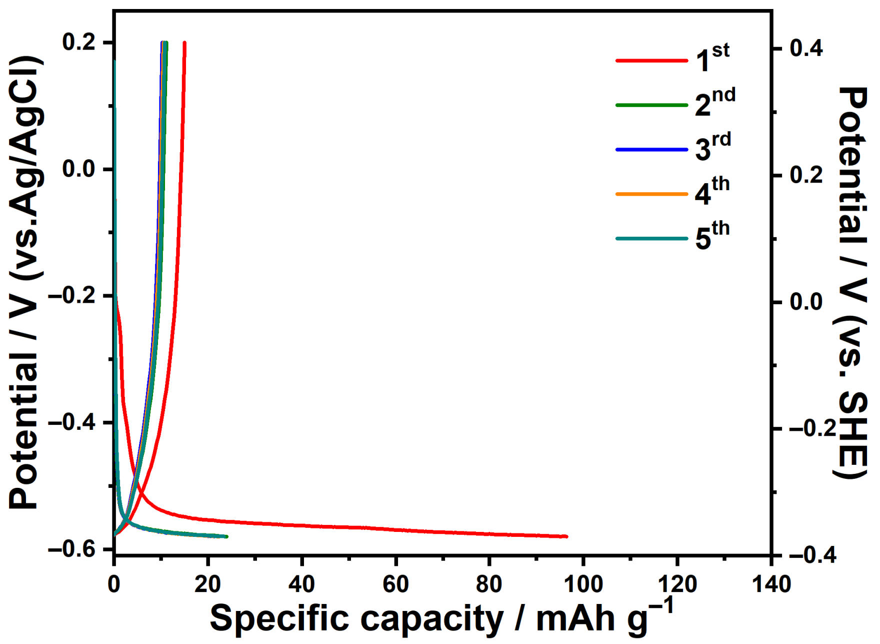

Supplementary Figure 6. GCD curves of a graphite electrode between –0.58 and 0.2 V *vs.* Ag/AgCl at a current rate of 100 mA g^{-1}.

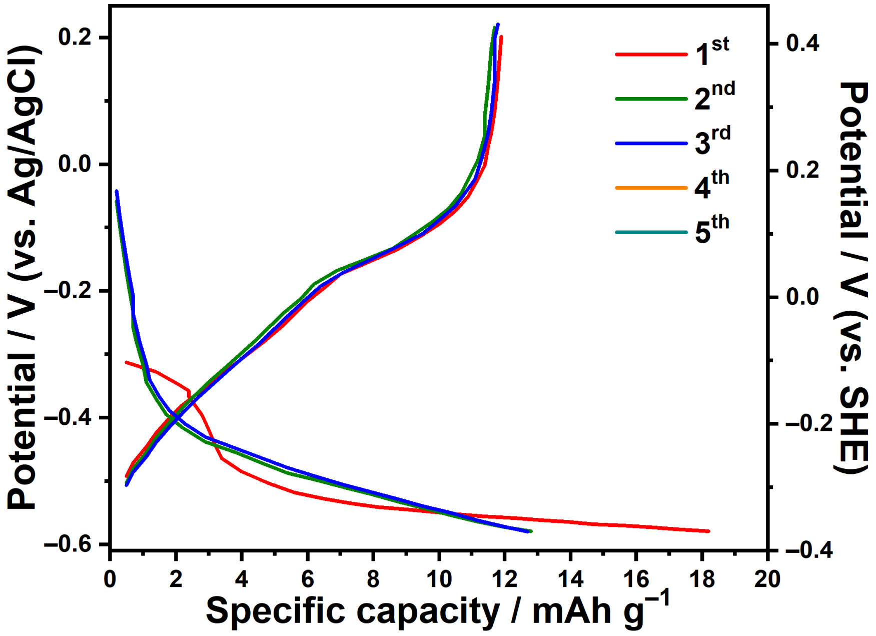

Supplementary Figure 7. GCD potential profiles of a PTCDA electrode in the diluted H_2SO_4 with pH = 3.5 at 100 mA g^{-1}.

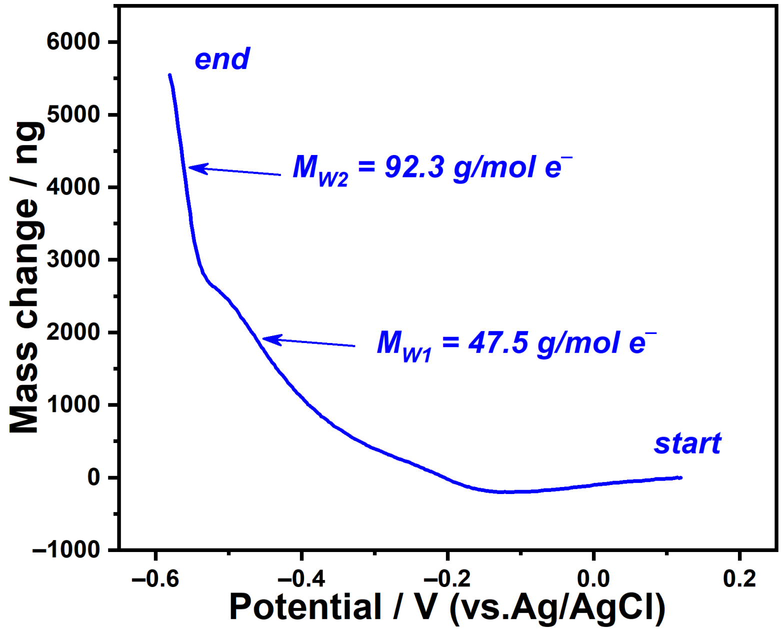

Supplementary Figure 8. EQCM curve recorded during a typical cathodic CV scan, showing the relationship between mass change and potential.

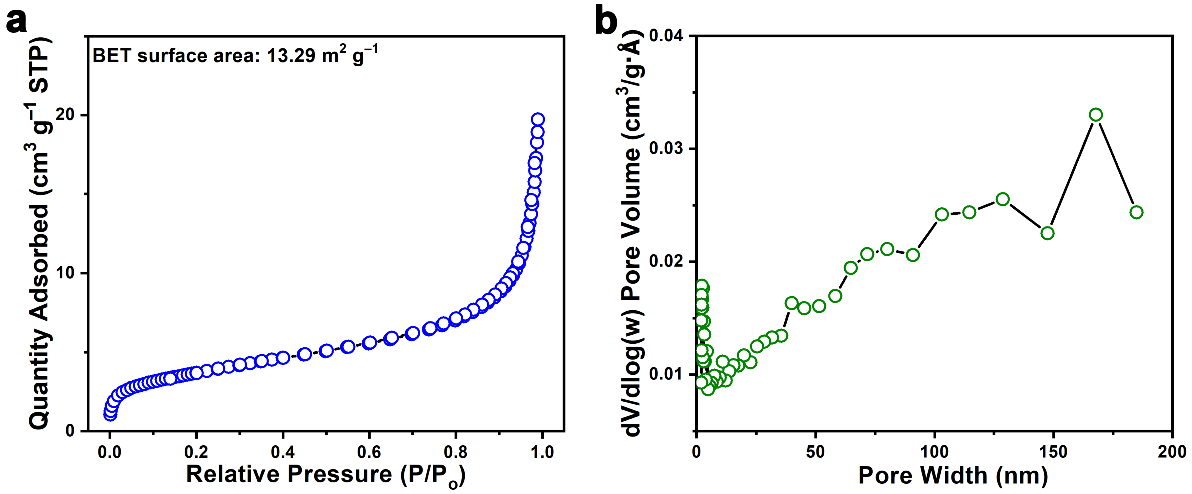

Supplementary Figure 9. Porosity analysis of PTCDA powder. (a) Nitrogen adsorption-desorption isotherms and (b) Pore size distribution of PTCDA.

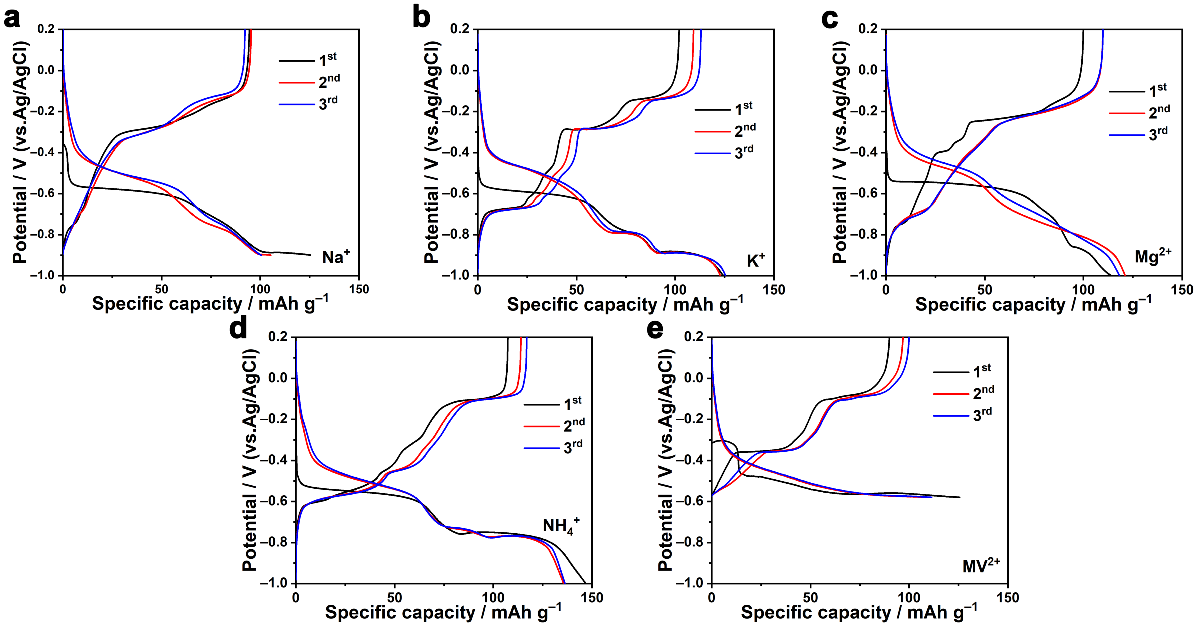

Supplementary Figure 10. Comparison of the GCD potential profiles for the storage of different ions in PTCDA at a current rate of 100 mA g^{-1}. (a) Na^{+}, (b) K^{+}, (c) Mg^{2+}, (d) NH_4^{+} and (e) MV^{2+}.

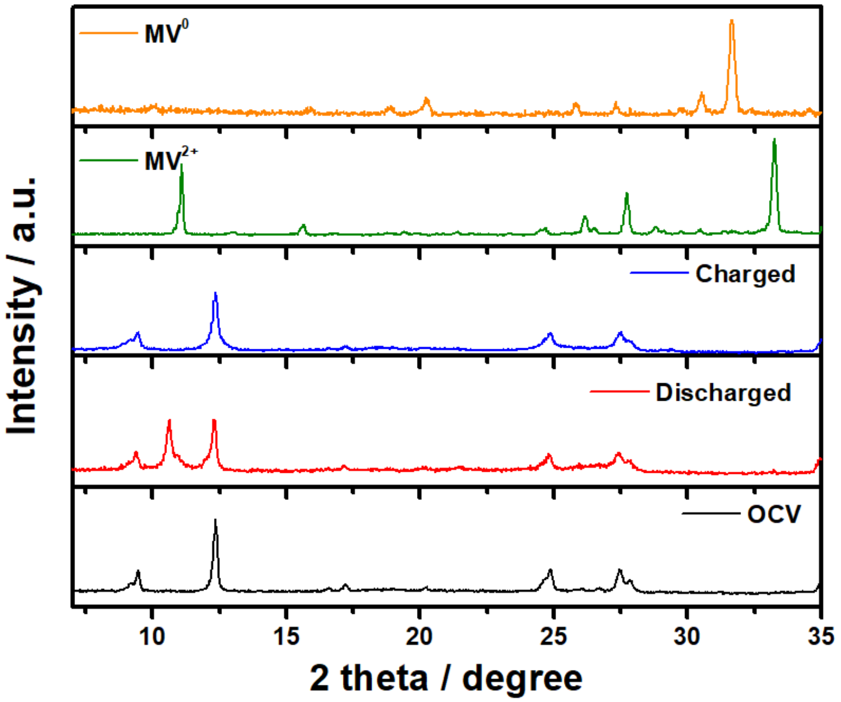

Supplementary Figure 11. XRD patterns of the PTCDA electrode recorded at OCV (black), the initial discharged (red) and the following charged state (blue); XRD patterns of methyl viologen dichloride powder (green) and its radical MV^0 (orange).

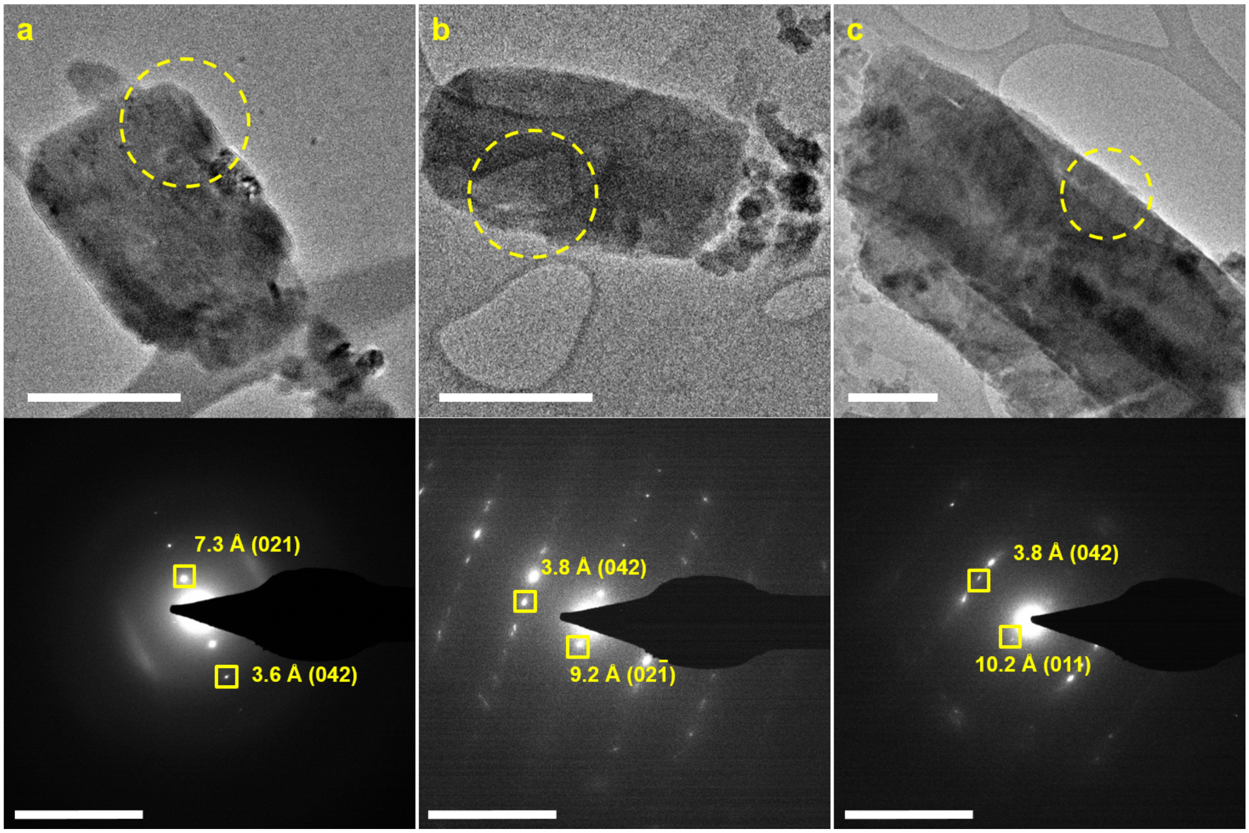

Supplementary Fig. 12. ***Ex situ*** **TEM images and SAED patterns of the PTCDA electrode at different state of charge.** (a) OCV; (b) Discharged to –0.58 V; (c) Charged to 0.2 V. Scale bars: top 500 nm, bottom 5 1/nm.

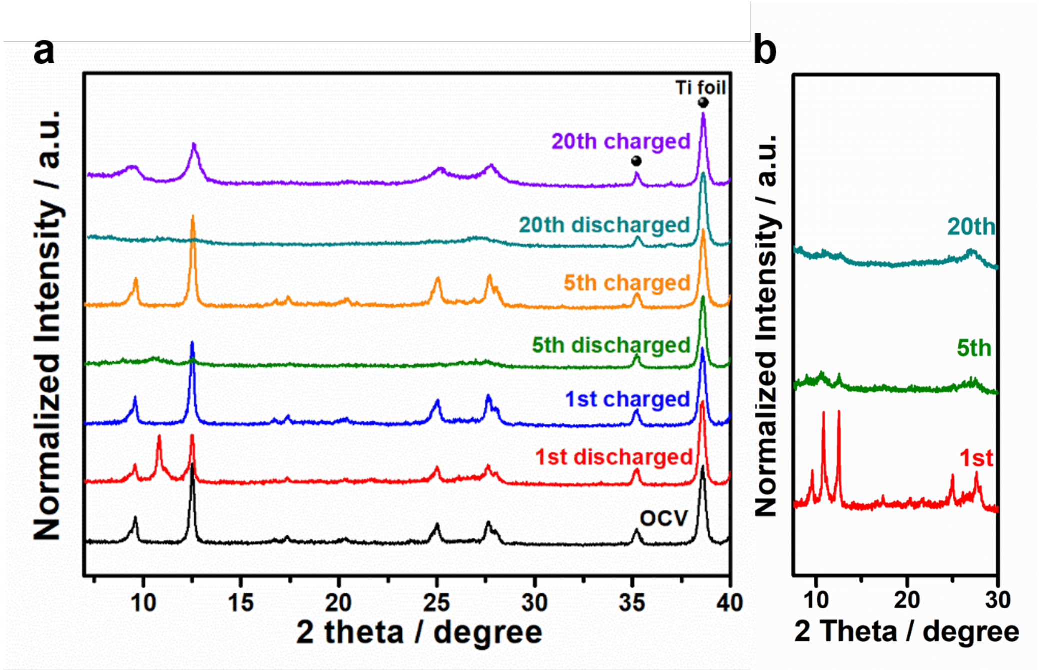

Supplementary Figure 13. ***Ex situ* XRD patterns for the PTCDA electrode at different state of charge.** (a) *Ex situ* XRD patterns of the PTCDA electrodes at OCV, the 1st cycle, the 5th cycle, and the 20th cycle; (b) Magnified *ex situ* XRD patterns of the PTCDA electrodes at discharged state in different cycles.

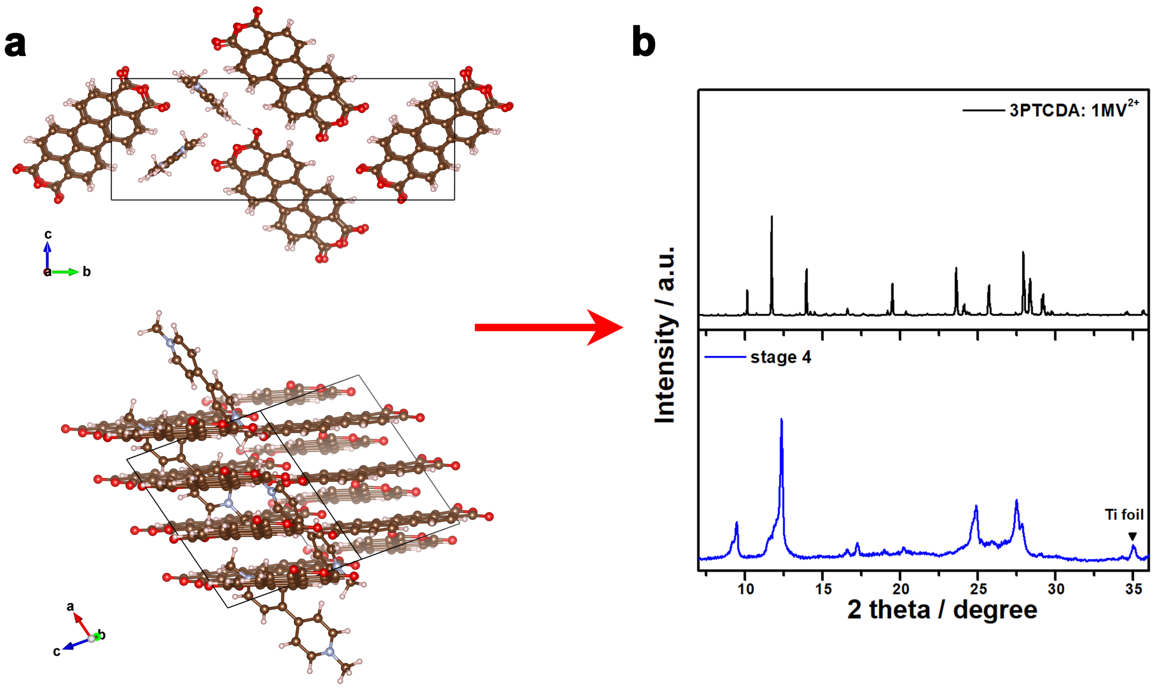

Supplementary Figure 14. The 45° vertical insertion model of a partially-filled PTCDA model (3 PTCDA: 1MV^{2+}). (a) The geometry after relaxation; (b) The corresponding XRD pattern.

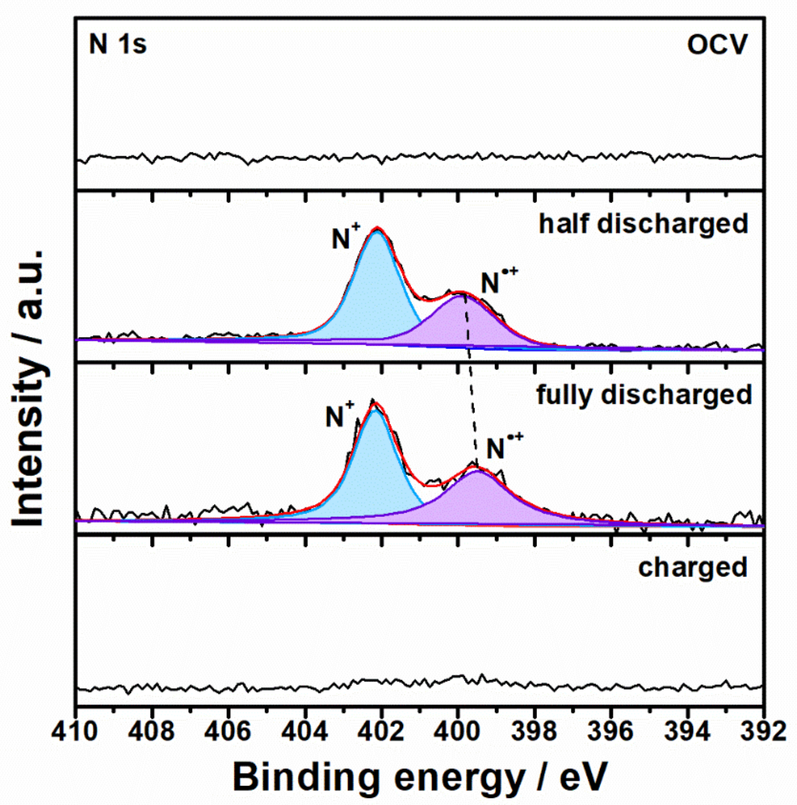

Supplementary Figure 15. XPS N 1s spectrum of PTCDA electrodes recorded at different state of charge. The N 1s spectrum of PTCDA at half discharged state could be fitted into two well-resolved peaks at 402.25 eV and 399.8 eV, which can be assigned to the positively charged nitrogen (N^{+}) and the cation radical ($N^{\bullet+}$), respectively.[1,2] The radical signal exhibited a shift toward lower binding energy from the half discharge state to the fully discharge state, suggesting that the methyl viologen got reduced after intercalating into the PTCDA structure. We are aware that, since the viologen dications can be reduced in the XPS analysis chamber during X-ray excitation to form viologen radical cation ($N^{\bullet}$),[2-4] we mainly use the results here as an approximate qualitative measurement.

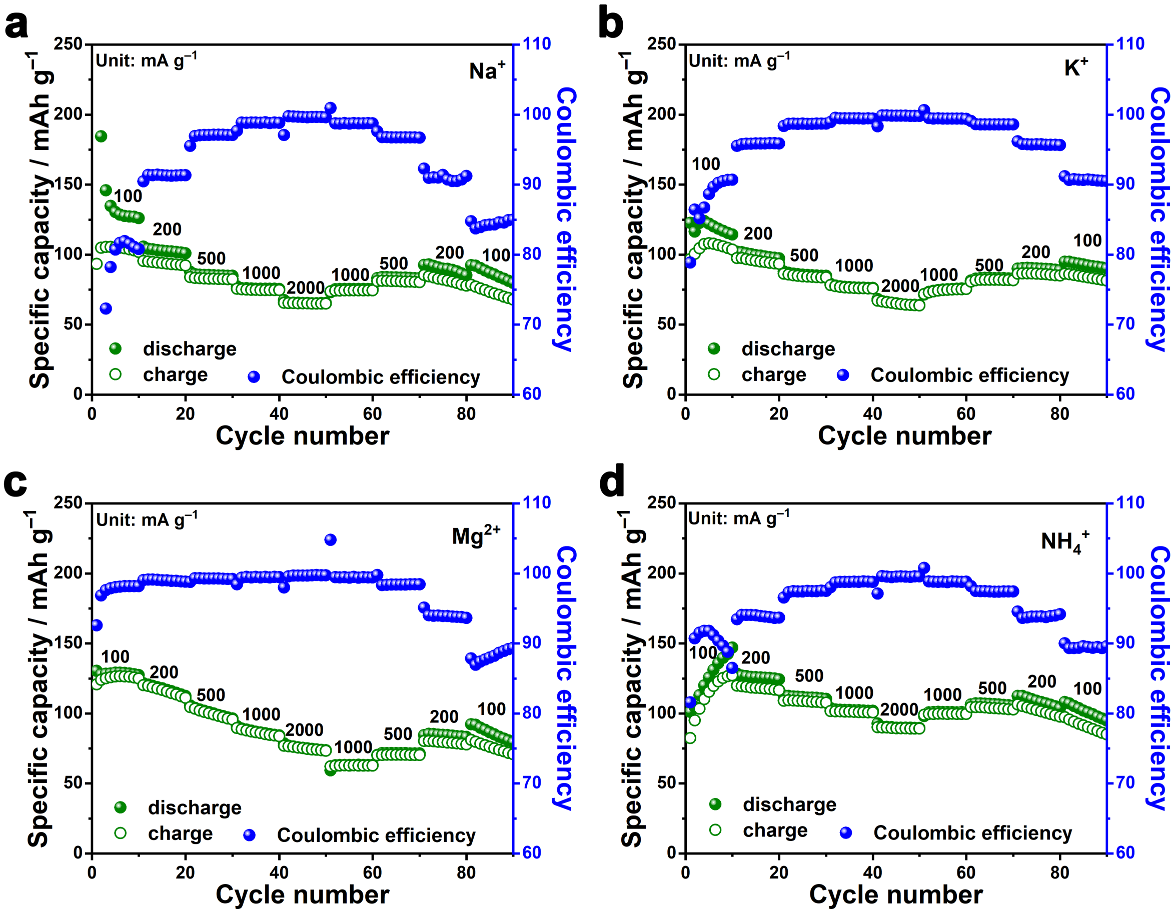

Supplementary Figure 16. Rate capability tests for the storage of Na^+, K^+, Mg^{2+}, NH_4^+ and MV^{2+} in PTCDA at different current rates.

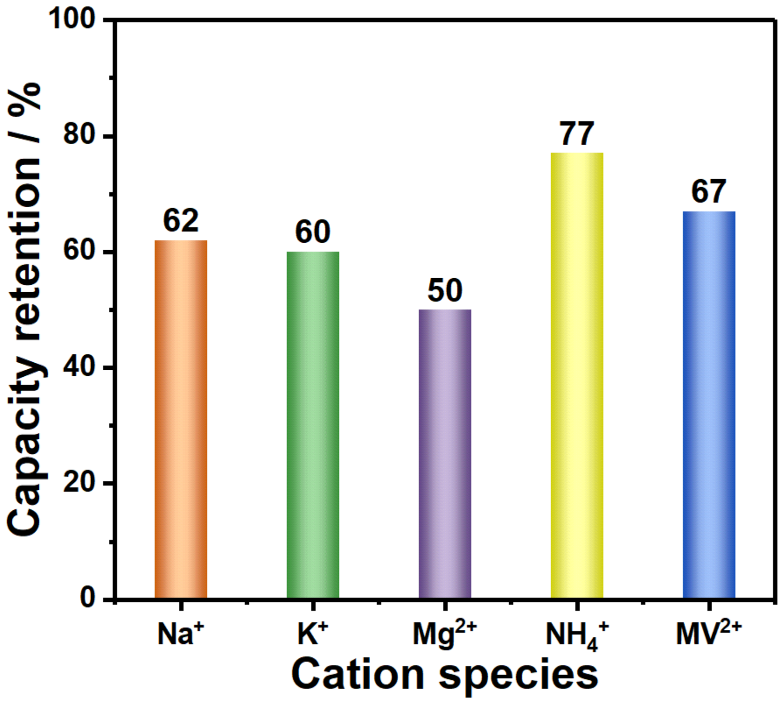

Supplementary Figure 17. The histogram illustrating the capacity retention of PTCDA in different battery systems during the current rates increasing from 100 mA g^{-1} to 2 A g^{-1}.

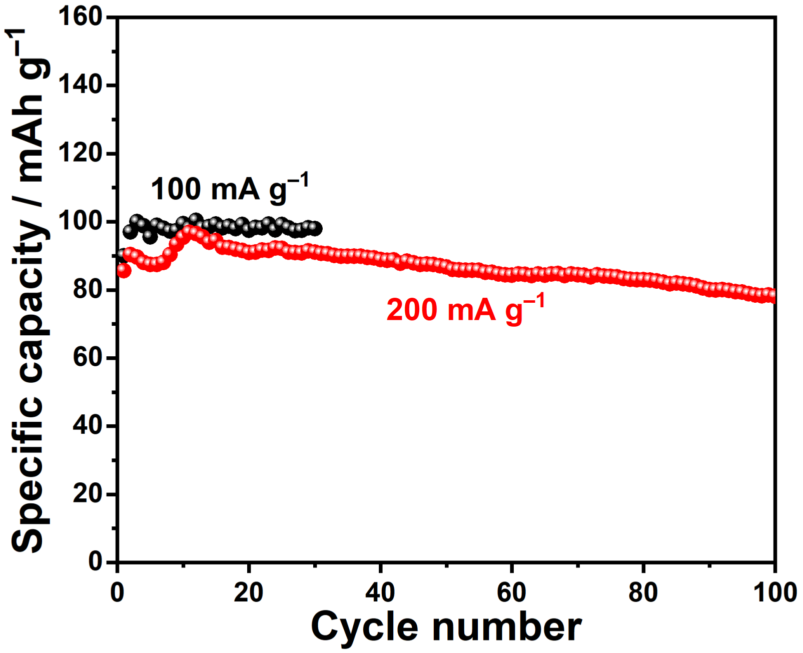

Supplementary Figure 18. Cycle performance of PTCDA at 100 mA g^{-1} and 200 mA g^{-1}.

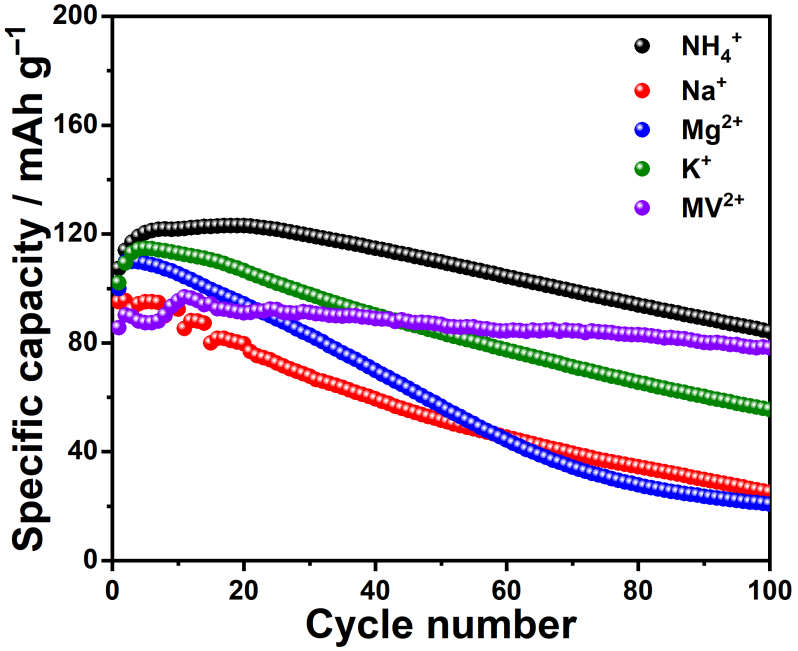

Supplementary Figure 19. Cycling stability tests for the storage of Na^+, K^+, Mg^{2+}, NH_4^+ and MV^{2+} in PTCDA.

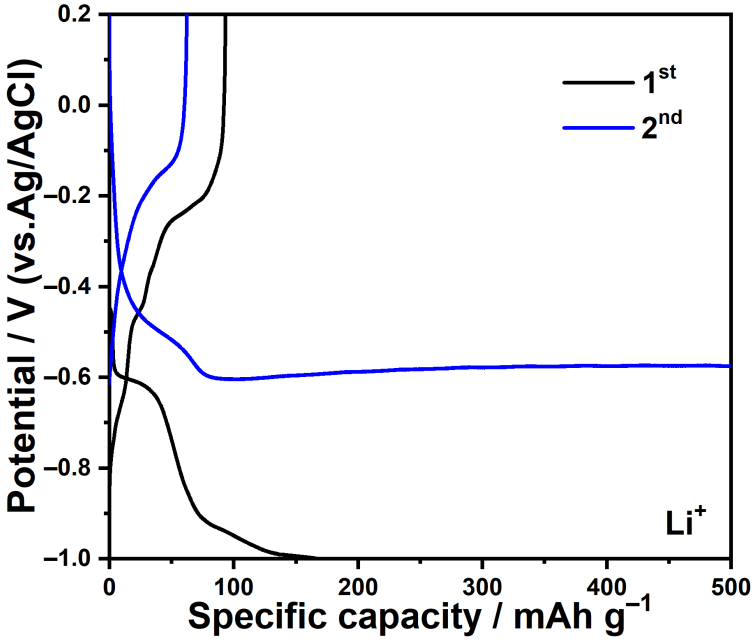

Supplementary Figure 20. GCD potential profiles for the storage of Li^{+} in PTCDA at a current rate of 100 mA g^{-1}.

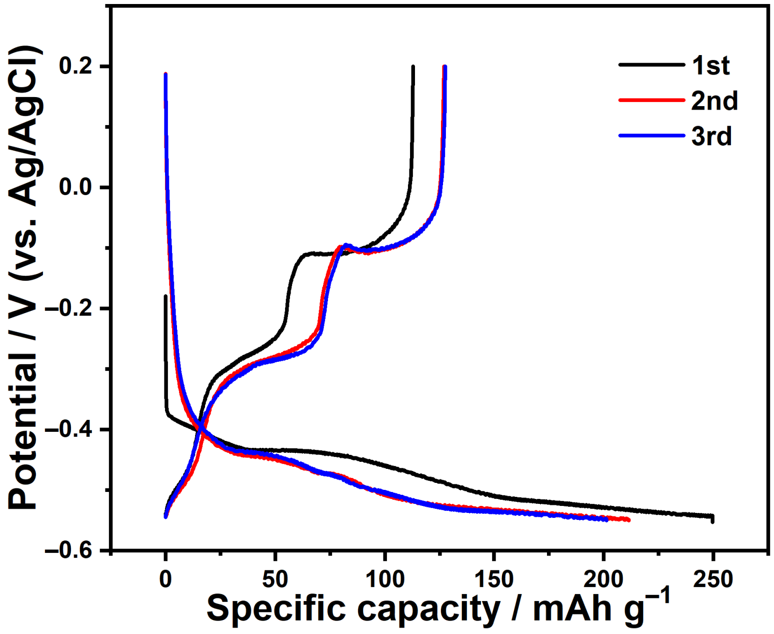

Supplementary Figure 21. GCD potential profiles for the storage of ethyl viologen in PTCDA at a current rate of 100 mA g^{-1}.

Supplementary Table

Supplementary Table 1. CHN elemental analysis results of the pristine electrode, the discharged electrode and the re-charged electrode.

	C (%)	H (%)	N (%)
Pristine	74.49	2.411	0.55
Discharged	70.19	3.363	2.58
Charged	74.36	2.335	0.70

Supplementary Note

Calculation detail of the ratio between MV^{2+} and PTCDA from the CHN element analysis results. The carbon in the discharged electrode consists of three parts: 70 wt.% active material (PTCDA-MV^{2+} complex, $C_{24}H_8O_6 + xC_{12}H_{14}N_2$); 20 wt.% conductive carbon (C-45, 100 wt.% C); 10 wt.% binder (PVDF, $-(CH_2\text{-}CF_2)_n-$, 37.5 wt.% C)

Hence, the weight ratio of carbon in the active material is: 70.19%–20%–3.75% = 46.44%. On the other hand, comparing with the pristine electrode, the weight ratio of the additive nitrogen that MV^{2+} brings into the complex is 2.03%, giving rise to a mass ratio of 46.44%: 2.03% = 22.9: 1 between carbon and nitrogen. (Note that the nitrogen in the pristine sample is attributed to the trace of air during the measurement.)

Then, combining the atom ratio of carbon ($24+12x$) and nitrogen ($2x$) with the mass ratio above, the molar ratio between the inserted MV^{2+} (x) and PTCDA is determined to be 0.579: 1.

Supplementary References

1. Gadgil, B., Damlin, P., Viinikanoja, A., Heinonen, M. & Kvarnström, C. One-pot synthesis of an Au/Au_2S viologen hybrid nanocomposite for efficient catalytic applications. *J. Mater. Chem. A* **3**, 9731-9737 (2015).
2. Alvaro, M., García, H., García, S., Marquez, F. & Scaiano, J. C. Intrazeolite photochemistry. 17. zeolites as electron donors: photolysis of methylviologen incorporated within zeolites. *J. Phys. Chem. B* **101**, 3043-3051 (1997).
3. Liu, X., Neoh, K. G. & Kang, E. T. Redox-sensitive microporous membranes prepared from poly(vinylidene fluoride) grafted with viologen-containing polymer side chains. *Macromolecules* **36**, 8361-8367 (2003).
4. Sampanthar, J. T., Neoh, K. G., Ng, S. W., Kang, E. T. & Tan, K. L. Flexible smart window via surface graft copolymerization of viologen on polyethylene. *Adv. Mater.* **12**, 1536-1539 (2000).
